# Single-cell transcriptome analysis for cancer and biology of the pancreas: A review on recent progress

**DOI:** 10.3389/fgene.2023.1029758

**Published:** 2023-04-06

**Authors:** Mona Tamaddon, Mostafa Azimzadeh, Peyman Gifani, Seyed Mohammad Tavangar

**Affiliations:** ^1^ Chronic Diseases Research Center, Endocrinology and Metabolism Population Sciences Institute, Tehran University of Medical Sciences, Tehran, Iran; ^2^ Department of Medical Biotechnology, School of Medicine, Shahid Sadoughi University of Medical Sciences, Yazd, Iran; ^3^ Medical Nanotechnology and Tissue Engineering Research Center, Yazd Reproductive Sciences Institute, Shahid Sadoughi University of Medical Sciences, Yazd, Iran; ^4^ Stem Cell Biology Research Center, Yazd Reproductive Sciences Institute, Shahid Sadoughi University of Medical Sciences, Yazd, Iran; ^5^ AI VIVO Ltd., Bioinnovation Centre, Cambridge, United Kingdom; ^6^ Genetic Department, Institute of Systems Biology, University of Cambridge, Cambridge, United Kingdom; ^7^ Department of Pathology, Shariati Hospital, Tehran University of Medical Sciences, Tehran, Iran

**Keywords:** single-cell sequencing, transcriptome analysis, pancreas biology, pancreas cancer, genetic heterogeneity

## Abstract

Single-cell sequencing has become one of the most used techniques across the wide field of biology. It has enabled researchers to investigate the whole transcriptome at the cellular level across tissues, which unlocks numerous potentials for basic and applied studies in future diagnosis and therapy. Here, we review the impact of single-cell RNA sequencing, as the prominent single-cell technique, in pancreatic biology and cancer. We discuss the most recent findings about pancreatic physiology and pathophysiology owing to this technological advancement in the past few years. Using single-cell RNA sequencing, researchers have been able to discover cellular heterogeneity across healthy cell types, as well as cancer tissues of the pancreas. We will discuss the new immunological targets and new molecular mechanisms of progression in the microenvironment of pancreatic cancer studied using single-cell RNA sequencing. The scope is not limited to cancer tissues, and we cover novel developmental, evolutionary, physiological, and heterogenic insights that have also been achieved recently for pancreatic tissues. We cover all biological insights derived from the single-cell RNA sequencing data, discuss the corresponding pros and cons, and finally, conclude how future research can move better by utilizing single-cell analysis for pancreatic biology.

## 1 Introduction

What if we knew which genes are specifically expressed in individual cells of the pancreas? This is a question an enthusiastic researcher would ask not long ago, with a vision of a possible method. Today, however, this question seems funny, owing to the development and speed of next-generation sequencing. We are now able to characterize a piece of desired tissue and dig into the individual cells and see which, for example, RNAs are expressed where. Being able to do the same for the whole genome, we are currently able to molecularly characterize single cells in tissues. To know the meaning of characterization, let us consider the matter of pancreatic tissue. By means of single-cell RNA sequencing, researchers are able to subtype the pancreatic tissue in more detail, find new immune-prominent cells and lineages, dig into tumor heterogeneity, trace different lineages toward development, and subtype various kinds of cancer and other pancreatic disorders, such as diabetes.

In this paper, we are going to review recent single-cell studies on the pancreas with a focus on RNA sequencing in a classified manner. To clarify, the reader will discover how single-cell RNA sequencing has helped us gather additional information on pancreatic biology. Therefore, we will narrate the surprising biological findings and compare and integrate the efforts in order to cast a light for future studies. The major part of this paper focuses on cancer similar to the literature; however, surprising findings about stem cells, new lineages in pancreatic normal tissue, efforts to characterize and map the overall pancreas, immunological studies and insights for the future are also discussed. We will incorporate the real data from selected landmark studies in order to provide a detailed comparative insight for the reader of the whole paper, which is not carried out in similar prestigious reviews in the field (Luo et al., 2020; Musa, 2020; Pompella et al., 2020; Han et al., 2021; Hematol et al., 2021; Mannarapu et al., 2021).

**FIGURE 1:**
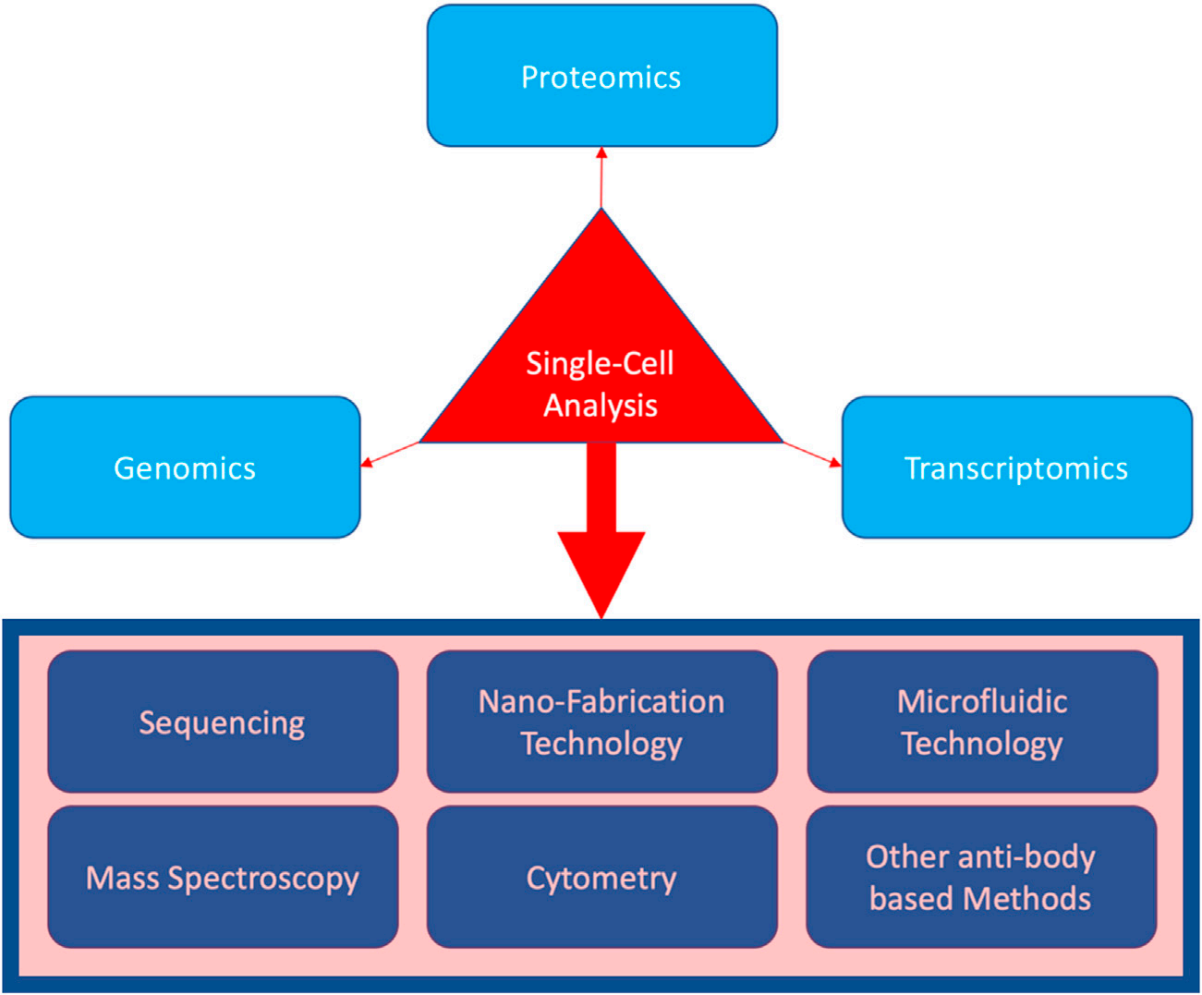
Three main biomolecular areas covered by single-cell analysis (in light blue), as well as the techniques incorporated (in the rectangular box).

It is also important to observe that single-cell analysis is not limited to RNA sequencing or even sequencing overall. Figure 1 shows different techniques have been incorporated in all the main areas of single-cell analysis. Notably, nano-fabrication technology is the main progressive core for carrying out the single-cell research as all the modalities, such as cytometric devices and sequencing technologies, are built upon an intricate nano-technology. However, it is only through sequencing that an unbiased study of all the possible genes in charge of a biological mechanism becomes available. To recapitulate, other methods such as cytometry and microfluidic sorting are dependent on biological reagents that are all chosen based on previous biological knowledge, while sequencing can provide the data for the whole genome or transcriptome, allowing us to look for completely new mechanisms involved. However, all these methods came with their own pros and cons, as for instance, sequencing will not be able to provide wide proteomic data and this suggests using combinatory methods for a thorough scientific endeavor. Good examples of such combination are new technologies such as single-cell sequencing assay for transposase-accessible chromatin (scATAC-seq) (Ji et al., 2020), which provides noisy but analyzable data on the regulome for the whole genome, invading both genomic and transcriptomic landscapes, and single-cell bisulfite sequencing (scBS-seq) (Clark et al., 2017), which provides genome-wide cytosine methylation data, processed to generate information on the transcriptome (Smallwood et al., 2014).

## 2 Single-cell sequencing for pancreatic cancer studies

Single-cell sequencing can provide data on genetic mutations, transcription status, and level of intensity for susceptible genes, responses to therapy, immunological responses, and overall tumor microenvironment. In addition to diagnosis and therapy, this technology can also bring us knowledge on relying causes and molecular mechanisms of cancer, especially by combinatory methods like some of our suggestions for future work throughout the paper. In this manner, in order to classify the literature, we first discuss the prototype studies providing insights into the pancreatic tumor microenvironment both pathologically and immunologically using single-cell RNA sequencing. Since invasiveness is regarded as one of the most distinctive features of cancer incidence, we review the studies reporting discoveries of new mechanisms for cancer progression using the technology. Finally, as cancer incidence is becoming significantly more diverse in the language of personalized medicine, we review the studies using the sequencing technology to put tumor heterogeneity in a molecular perspective. However, let us first start with a discussion on the disease itself.

### 2.1 Pancreatic cancer

Pancreatic cancer has received significant attention from biomedical researchers worldwide. According to the American Cancer Society, most of the incidents of pancreatic cancer originated from the central tissues and are, therefore, called exocrine cancers, a great deal of which are pancreatic adenocarcinomas that originate from duct cells (Chen et al., 2021b). However, there are less prevalent subtypes, with the most famous being the one that originates from the acinar cells and is called the acinar cell carcinoma. Overall, there are endocrine incidents of pancreatic cancer called the pancreatic neuroendocrine tumors (NETs), or islet cell tumors. They are significantly less prevalent (less than 2% of pancreatic cancers); however, they have greater prognostic capability (Marini et al., 2021). Not surprisingly, most cancers originate from regions that had the stem cells present. To be more precise, cells with regenerative capabilities were uniquely found at the central ducts and exocrine parts of the pancreas. Similarity and proximity of genes for cancer prognosis and stemness are important to understand the prominent cause of this correlation. Both cancer prognosis and stemness are based on maintaining the mitotic capability in a specialized molecular way, which remains a great substrate to build our interpretation of genes contributing in both. This is exactly where single-cell transcriptome studies of the pancreas can become helpful in navigating us through complex molecular biology of both cancer and stem cell research. Hence, we begin by discussing pancreatic cancer research using the tool of single-cell RNA sequencing, while casting a glance on insights for non-cancer research.

### 2.2 Insights into the tumor microenvironment

Numerous groups around the world are focusing on one of the greatest factors affecting the tumors’ faith in the body, i.e., the microenvironment they live in (Khatami and Tavangar, 2018; Khatami et al., 2018; Khatami et al., 2019; Raghavan et al., 2020; Shabani et al., 2020; Chen et al., 2021a). Ligorio et al. (2019) reported how the stromal microenvironment could navigate the intra-tumoral architecture. They managed to reconstruct the tumor tissue and identify eight subgroups according to the architecture of the tumor gland. As they conclude, cancer-associated fibroblasts play a key role in shaping cancer cells’ microenvironment and, hence, the tumoral architecture. Reshaping the tumor architecture is not limited to one group, as for instance, Moncada et al. (2020) also attempted a tumor molecular configuration reconstruction using the microarray technology and single-cell sequencing. In order to cast light on the novelty of this type of research, we discuss the journey from the beginning, as it all started with the birth of spatial transcriptomics (ST) in science (Vickovic et al., 2014). With normal transcriptomics, we would have the molecular data for a pool of cells from a piece of tissue, out of which different lineages can be identified using their molecular attributes. However, with spatial transcriptomics, cell clusters are analyzed on microarrays that can serve as a vector to the position of the analyzed cluster. Hence, molecular reconstruction of the whole tissue architecture becomes available. Notably, ST does not offer a single-cell resolution, and Moncada et al. only attempted to push the limitation line forward as the combined ST data with single-cell RNA sequencing provided single-cellular details of the ST cell clusters. In other words, future research for spatial transcriptomics must rely on the way of increasing the resolution of analytical microarrays in order to enhance the reconstruction output resolution to a single-cell level, just as Moncada et al. (2020) attempted. Also, a combination of this technology with other convenient methods seems beneficial. A good example of such combination is the recent paper by Cui Zhou et al. (2022) that used single-cell RNA sequencing in collaboration with spatial transcriptomics, bulk proteogenomics, and cellular imaging. By mapping mutations and copy number events, they were able to notify normal cells and distinguish cells with multiple neoplasia. They also combined histology and spatial transcriptomics to identify the transitional subpopulations. To enlighten, they used histopathological data for deconvolution of ST data. This method is suitable for finding several gene expression relations as they discovered for TIGIT and Nectin in regulatory T cells and tumor cells, respectively (Cui Zhou et al., 2022).

On the other hand, sequencing in combination with modern genetic edition technologies has enabled us to molecularly monitor every single desired change in the microenvironment of any cell. To enlighten with an example, Chen et al. (2021c) deleted type 1 collagen in the myofibroblasts surrounding the pancreatic cancer cells and monitored the changes in the immune response and the progression of cancer. Meaningfully, they made the fibroblasts surrounding the tumor dysfunctional by eliminating their most abundant collagen type. Their results suggest that therapeutic methods severely interfering with the metabolism and molecular well-being of fibroblasts and healthy tissues surrounding the tumor site would be favorable for disease progression or possible reemergence of tumors if we are not successful in targeting all the cancer stem cells.

**FIGURE 2:**
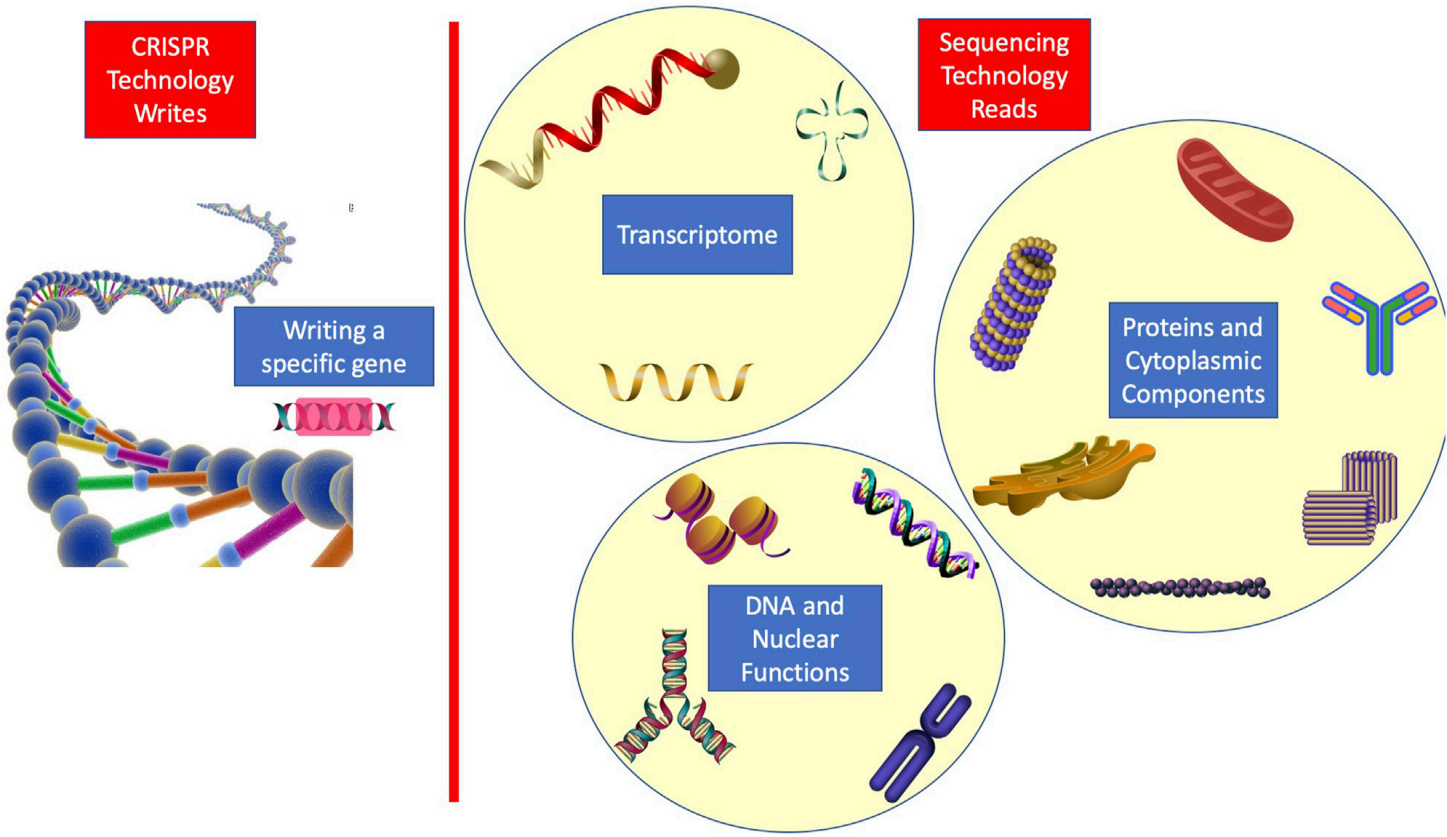
Illustration of how sequencing and CRISPR can be used in combination to further improve human knowledge on various biological functions.

In order to further enlighten the role of RNA sequencing in the research study, we discuss another modern therapeutic method that must rely on single-cell sequencing for monitoring the resulting changes. CRISPR engineering has enabled the single-cell genome edition with a significant precision level, and using this cellular therapy, *in vivo* precise genetic therapy is becoming available (Zhang, 2021; Zhang et al., 2021). Notably, it is through single-cell sequencing that we can verify the genetic changes we make and the resulting molecular effects with the greatest accuracy. To recapitulate, CRISPR enables us to write in a genetic language, while sequencing provides the reading in genetic and transcriptome wide languages. Hence, it is beneficial for future works to focus on writing particular genetic codes and trying to figure out changes all across the transcriptome, exploiting both technologies. Figure 2 shows the idea of how a wide range of biological functions can be viewed from a molecular perspective using the combination.

**FIGURE 3:**
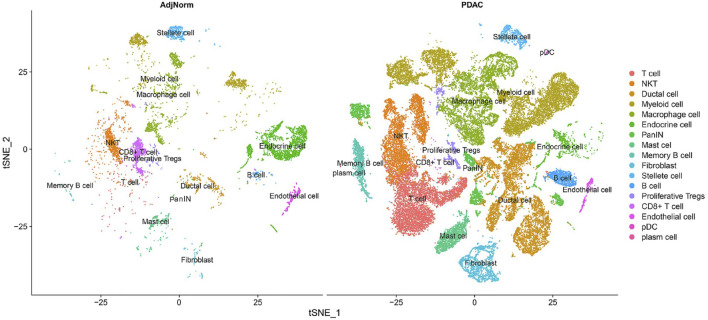
Characterizing all the immune cells in the pancreatic tumor microenvironment (Tang et al., 2021).

Moreover, single-cell data enables us to gather novel immunologic insights of cancer. For instance, Tang et al. (2021) performed a single-cell analysis on the tumor microenvironment to characterize the set of present immune cell types. As parts of their results are shown in Figure 3, all the present immune cells have been characterized and subset into populations matching the criteria of each particular category. The immunologic composition of the tumor microenvironment is of great importance, especially recently after numerous cross-talks and relationships between different immune cell types have been observed (Raghavan et al., 2020). Future therapy has no other way than to rely on these molecular findings regarding the tumor-immune microenvironment. Regarding the increasing immunological prominence in research, we discuss immunologic findings using single-cell RNA sequencing for the pancreas in the last section of this paper.

### 2.3 Discovery of new mechanisms of cancer progression

Although thorough knowledge of the underlying causes of pancreatic cancer and the molecular path from incidence to metastasis is yet to be investigated, some groups have gathered novel information on the matter using single-cell RNA sequencing. For instance, Zhu et al. reported a lethal molecular stage prior to invasiveness in pancreatic cancer (Id et al., 2021). To clarify, they have discovered the pre-invasive transition of adipose-derived stromal cells into *COL11A1*-expressing cancer-associated fibroblasts. With the transition being essential to invasiveness, this can be considered a discovery of a molecular target with therapeutic applications. In a similar finding, Dominguez et al. (2019) reported the transition of stromal cells into LRRC15 + myofibroblasts as the determinant of a patient’s response to cancer immunotherapy. Notably, monitoring these kinds of lineage transitions has become part of our everyday research as a result of single-cell RNA sequencing incorporation. Not surprisingly, here both groups have used single-cell sequencing; the first was on the tissues of patients at different stages of pancreatic cancer, and the second was on an animal model. Their common approach was to look for common molecular signatures in different types of cells at various stages of the progression but present in some new lineages emerging during cancer. To restate, first, the lineage in charge of progression and invasiveness was found, and then, using the molecular signatures present in the transcriptome, ancestor cell lines were discovered. Next the figured-out transition was monitored as a corroboration to be ensured. This methodology can be beneficial in cancer research as each group digs out a bit more of the underlying molecular mechanism in charge of the disease. However, for the benefit of patients, more combinatory research studies to figure out a molecular map for cancer progression are needed. A map is presented from which the best personalized criteria of molecular targets can be administered to the patient. The complexity of this map refers mostly to the fact that it is about unlimited correlations and cross-talks, not only at the RNA level but also at DNA levels, and all the functional proteins and even lipids in the cell. To enlighten this multilayer nature, Yue et al., for instance, shed light on the prominence of genetic variations in controlling the transcriptome evolution in cancer (Khatami and Tavangar, 2018; Chan-seng-yue et al., 2020), while Dey et al. (2020) discovered the role of cytokines in cell reprogramming during cancer courses.

Single-cell RNA sequencing has made a significant contribution in further classification of the diseases. In other words, sometimes, new molecular findings change the classical way we previously looked at the classification of diseases, such as cancer, similar to Chung et al.’s (2019) prominent finding. They reported the signaling between endocrine and exocrine pancreas to be in charge of molecular progression in PDAC. Notably, this changes our classic view of considering PDAC as an exocrine pancreatic disorder standing alone.

**FIGURE 4:**
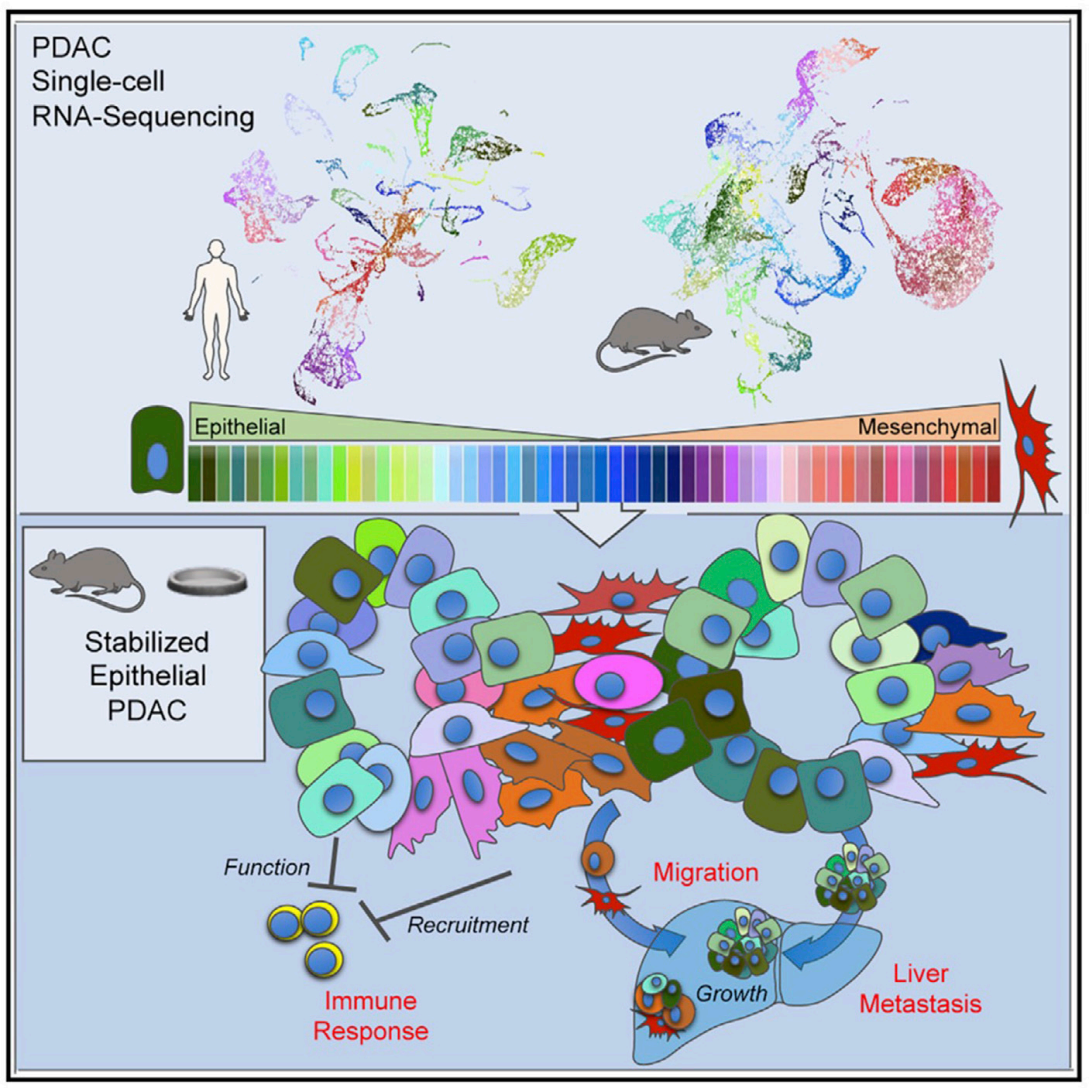
Carstens et al. (2021) provided evidence on how an epithelial phenotype of the primary tumors promotes metastasis and inhibits immune responses; they classified the primary tumors using single-cell RNA-seq.

One of the most important factors to be considered a significant help to pancreatic biology is the composition of modern genetic knockdown and single-cell sequencing. In other words, we are able to simultaneously alter the genome and monitor the effects at molecular and cellular levels. As a standard example for this kind of research, Shah et al. (2017) inspected in detail the molecular changes in APE1/Ref-1 knockdown. In this methodology, they took a gene of selection, knocked it down, and visited all the resulting molecular changes with sequencing to figure out the related pathways and genes with respective affinities. Sometimes, we know what the genes do and knock them down to observe the biological side effects in certain disorders such as cancer. Carstens et al. (2021), for instance, knocked *Snail*, *Twist*, and *ZEB1* genes to stabilize the tumor in the epithelial state. In all efforts, this ensured epithelial stabilization led to the promotion of cancer progression, immune suppression, and improvement in the liver’s metastatic conditions. Although epithelial to mesenchymal transition occurs during cancer, they claimed that stabilizing the tumor in the epithelial state promotes collective cell migration and results in colonized, hence more powerful, metastasis. The possibility of classification of tumors in the epithelial–mesenchymal spectrum, as they did in Figure 4, is all again owed to single-cell sequencing.

### 2.4 Tumor heterogeneity in a molecular perspective

One of the most prominent roles of single-cell RNA sequencing in the current research is to enable us to discover the traces of cell lineages even with a few cells of that lineage present in the tissue. To restate, a piece of tissue such as the pancreas, which was classically categorized and identified based on different macro compartments, such as ducts and islets, with their specific cells, can now be characterized down to single cells; this way, an avalanche of newly discovered lineages with possible roles in immunotherapy or regenerative medicine is in line.

**FIGURE 5:**
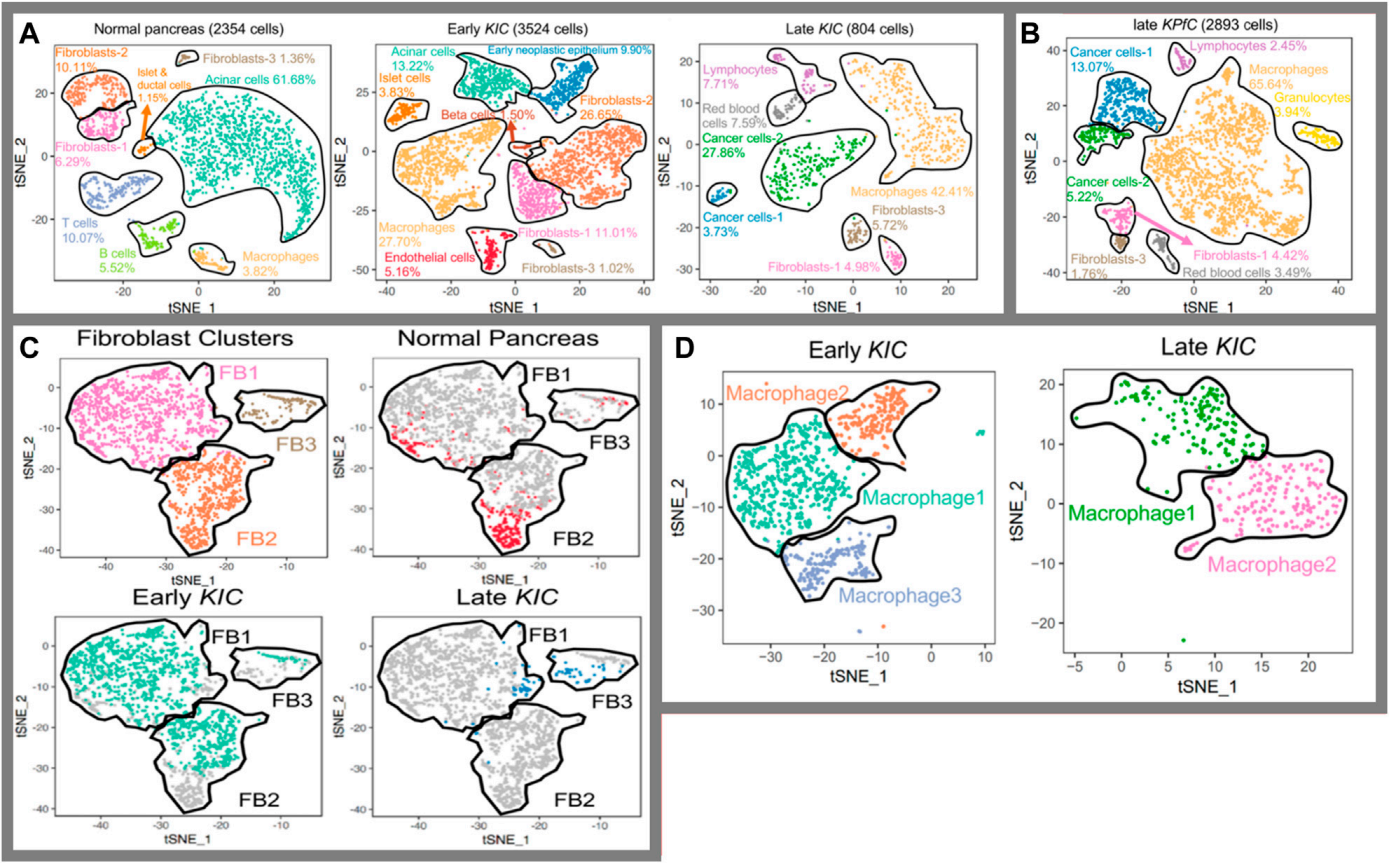
Identification of tissue composition at a molecular level (Hosein et al., 2019). (**A**) Cell populations in normal, early, and late cancerous tissue; **(B)** comparison in between two subtypes of KRAS-induced cancer; **(C)** fibroblasts into prospective in normal tissues as well as early and late cancer tissue,; and **(D)** populations of macrophages in magnification through the cancer course.

**FIGURE 6:**
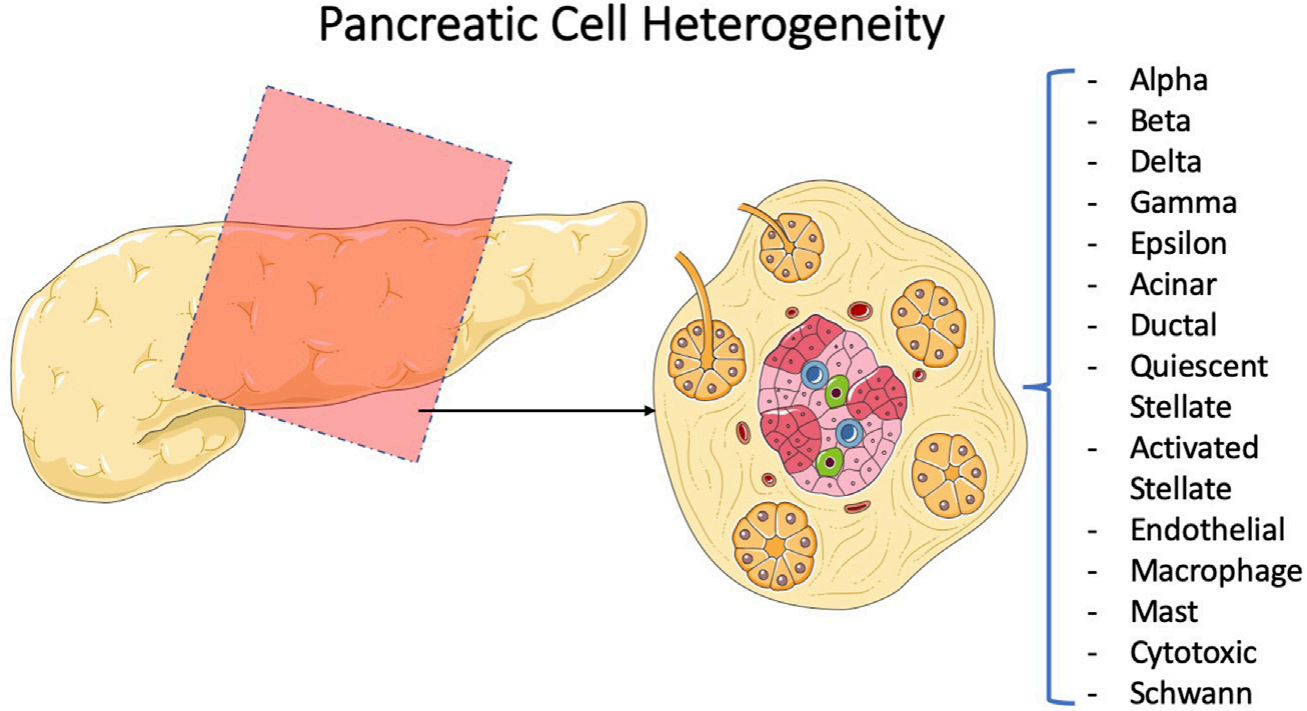
Efforts to characterize and subtype the pancreatic tissue. Comparing the percentages for each subpopulation of the pancreatic tissue under normal and disease conditions using sequencing technology has been the method of diagnosis for several groups. Baron et al. (2016)conducted a descriptive study on pancreatic cellular subtypes, and Wollny et al. (2016) shed light more on one subtype, namely, the diabetically prominent islets.

For pancreatic cancer, there have been several groups working on how single-cell RNA sequencing can reveal tumor heterogeneity (Quandt et al., 2011; Peng et al., 2019; Schlesinger et al., 2020). Since the output is more detailed molecular data on tumor cell types, this type of research is favorable for personalized medicine, where particular therapy specialized for an individual is prescribed. More detailed data lead to more detailed classifications and that leads to more specialized classification of patients on the road to personalized medicine. To recapitulate, single-cell RNA sequencing can surely alter the way we categorize various diseases in the future, illuminating their molecular particularity. In this regard for pancreatic cancer, groups focus on descriptive studies to unveil the disease at a molecular level (Sho et al., 2017; Lin et al., 2020; Ren et al., 2021). They have all used pieces of cancerous or surrounding normal tissues to perform single-cell RNA sequencing and attempted to classify the cellular population based on discrepancies in transcriptome (Lin et al., 2020; Tamaddon et al., 2022). This categorization-based methodology of research can be undertaken in different orders of style. Hossein et al., for instance, took a stepwise approach in which they initiated the categorization into macro-types using the main molecular traces (i.e., all the fibroblasts into one category) (Hosein et al., 2019). They carried out the categorization for the early and late stages of progression and comparatively two distinct tumor subtypes. Then, they stepped up to navigate different subpopulations of fibroblasts and macrophages as two key elements affecting the tumor faith (Figure 5). To criticize this type of research, it is important to notice that single-cell RNA sequencing data from incidents of cancer in pieces of cancer tissue from mice cannot be considered the standard for the cancer progression phenomenon. However, it provides a strong basis for future similar research on human tissues and cross species in order to figure out the molecular path of incidence and progression for pancreatic cancer. Also, it is not limited to disease conditions as it can also be beneficial in increasing our knowledge of healthy pancreatic subpopulations. For instance, Baron et al. (2016) conducted a thorough research and an immense single-cell analysis to provide the transcriptomic map of human and mouse pancreases. They tried to reconstruct the overall pancreatic tissue based on 14 distinct cellular types. Efforts like these can navigate human beings’ knowledge on precision anatomy. Also, this type of research is complementary to the style of Wollny et al. (2016) as they tried to subtype pancreatic islets. In other words, they took one more step toward the architecture of islets. The idea for all these types of research is shown in Figure 6, and the method provides a detailed image of pancreatic tissues.

## 3 Pancreatic pathology, for abnormalities other than cancer

Applications of single-cell sequencing in pancreatic studies for sure are not limited to cancer. CP or chronic pancreatitis, for instance, was the topic of interest of Mao et al. (2022) as they characterized the ductal cell in the process of the disease. Their study is a good example of how a set of genes and markers responsible for a condition could be revealed in a single study. With clustering methods, they were able to sub-populate the cells and carried out precise genetic analysis to discover few hundred upregulated and downregulated genes. However, only overexpression for MMP7 and TTR was further verified. Other than CP, pancreatic disorders of various types such as acute (Sun et al., 2021) and chronic pancreatitis (Weiss et al., 2018) and diabetes have also been genetically investigated using the single-cell sequencing technology. Also, sequencing has shed light on our microbiome knowledge, enabling numerous groups to identify the relationship between various pancreatic disorders and gut microbiota with the help of sequencing (Ammer-Herrmenau et al., 2020; Qi-Xiang et al., 2022).

## 4 Single-cell RNA sequencing for pancreas biology

### 4.1 Multipotency in pancreatic tissue

**FIGURE 7:**
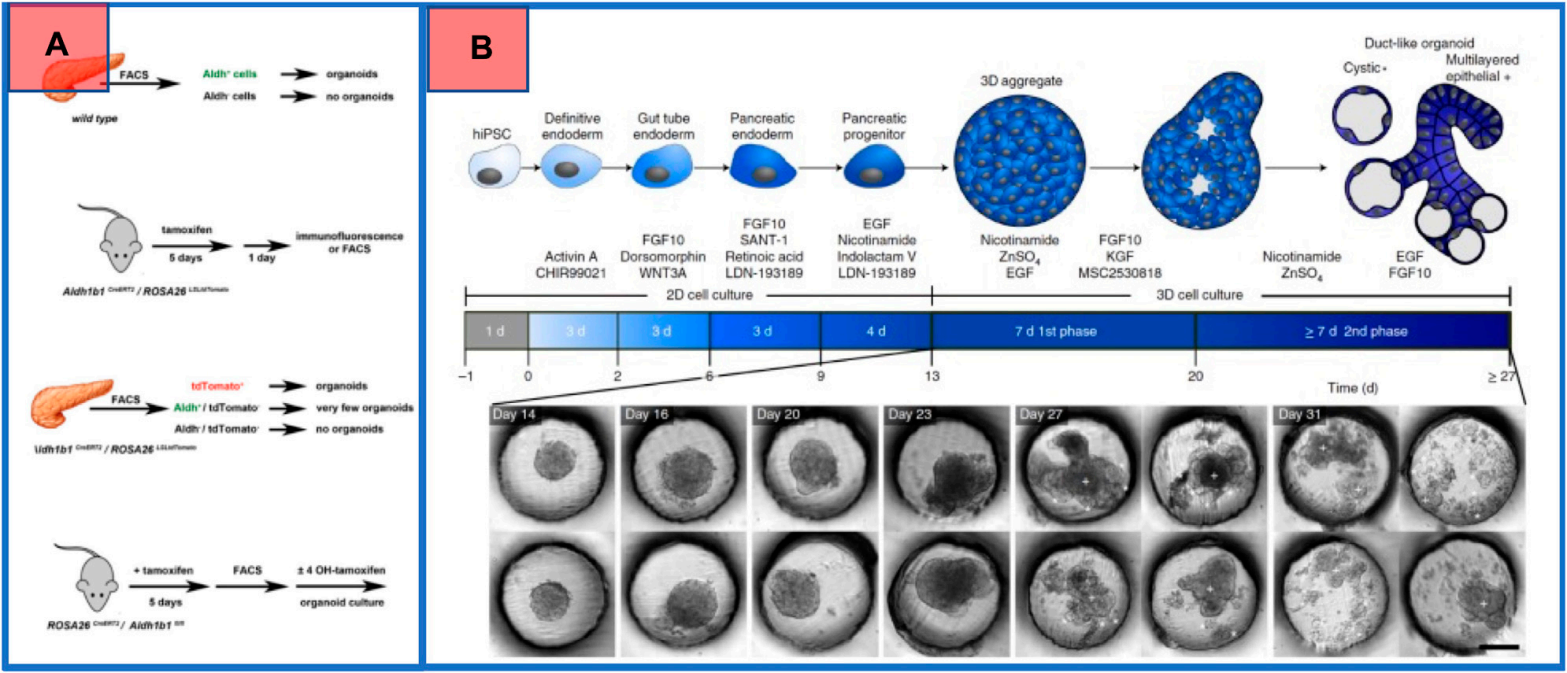
Effort to isolate and characterize human stem cells. The embryonic and adult stem cells have been characterized owing to single-cell RNA sequencing which now provides a substrate to look deeper for underlying mechanisms. **(A)** Wiedenmann et al. used microfluidic technology to grow duct-like organoids from pancreatic progenitors (Wiedenmann et al., 2021). **(B)** Mameishvili et al. provided evidence that *ALD1HB1* is the selector gene for pancreatic adult progenitors and is associated with KRAS-driven pancreatic cancer.

Multipotent cells with regenerative abilities have recently been an interesting topic in biomedical research. Finding new lineages with stemness in the tissue will significantly increase hopes for regenerative capabilities and therapeutic applications (Hutton et al., 2021). To enlighten the prominence, suppose we discovered multipotent cells in the pancreas with the capability to differentiate into beta cells under certain conditions, which could help in the fight against diabetes. In this regard, numerous groups reported to have discovered new progenitor-like cells and characterized the previously known progenitors to uncover pancreatic development. Krentz et al. (2018), for instance, characterized known progenitor cells from embryos of mice to derive beta cells and provided a detailed description of the pancreas at the molecular level during development in mice. This can be used as a guide for efforts to form beta cells in both vitro and vivo from human embryonic and adult stem cells. The presence of stem cells in the adult pancreas and their ability to evolve to functional beta cells have been controversial in research in recent years. However, in a comprehensive research conducted by Mameishvili et al. (2019) published in PNAS in 2019, the controversy for the presence of isolable stemness in an adult pancreas somehow ended. Since, a signature gene (*Aldh1b1*) has been reported to be a certain flag for isolating cells with progenitor behavior and the capability of evolving to all three main subtypes in the adult pancreas. Not surprisingly, the same gene is reported to be responsible and core to pancreatic cancer (performed particularly on the KRAS-induced subtype) (Mameishvili et al., 2019). Some groups have moved further, exploiting technology to form pancreatic organoids *in vitro* in order to molecularly monitor their differentiation. For example, Wiedenmann et al. (2021) used microfluidic technology and single-cell transcriptome monitoring to unveil the molecular path of differentiation from pancreatic progenitor cells to duct-like organoids. The combinatory in Figure 7 shows how discussed groups unveiled different parts of the mystery of pancreatic stemness biology.

### 4.2 Pancreatic fibroblasts into perspective

There have been numerous groups publishing papers focusing on fibroblasts as key to biological mechanisms they look for in the pancreas (Elyada et al., 2019; Kieffer et al., 2020; Steele et al., 2021). Fibroblasts are common cell types prevalent in various parts of the body and different tissues. Day by day, new insights into the effectiveness of fibroblasts on various molecular mechanisms of cancer progression and immune responses are being discovered (Sebastian et al., 2020). Therefore, we designated a separate part discussing which pancreatic fibroblast findings are most thankful for single-cell RNA sequencing technology.

Concluding based on the literature (Elyada et al., 2019; Kieffer et al., 2020; Hutton et al., 2021), it seems that according to pancreatic ductal adenocarcinoma that fibroblasts play a key role in mediating the immune microenvironment. Even new subtypes of fibroblasts are discovered every now and then. For instance, Hutton et al. (2021) has discovered a new classification of fibroblasts using the CD105 marker, and they could engineer how this fibroblast lineage affects tumor development. To restate, the CD105-negative fibroblasts supported anti-tumor immunity, while CD105-positive ones were tumor permissive. Finding lineages like this can be much beneficial in moving toward better therapeutic regimens. Notably, they used mass cytometry to carry out the single-cell analysis. This method is suitable for working with known markers by figuring out a new combination of markers like what they did. However, as Wang et al. (2021) did for the same purpose of subtyping the fibroblasts, single-cell RNA sequencing has a greater precision generally and provides much more information, the pool from which we can fish out new biomarkers. Finally, as we also discussed in the previous sections, fibroblasts significantly contribute to the condition for the tumor microenvironment and play an important role in tumor heterogeneity.

### 4.3 Pancreatic tissue biology

Monitoring single cells at the transcriptome level can gather much information on various corners of biology. Evolution and development are prominent corners, for instance. In this regard, Enge et al. (2017) did a good job of uncovering more of the molecular pattern of aging. They tried to uncover the genes in charge of the transcriptional noise inherent in aging. Their idea and results seem surprising. However, it is important to note that all the 2,544 cell sequences were carried out from samples taken only from eight donors, spanning 60 years of age. Hence, individual discrepancies at a single-cellular level seem to have an inevitable effect. Stepping up these kinds of studies to apply on tissues from numerous human or animal models can better verify the genes in charge of developmental or aging processes.

One other corner of the biological planet covered by single-cell sequencing in the pancreas is immunology. Now and then, new relationships between diverse types of immune cells and new lineages with particularly prominent functions are discovered (Pan et al., 2019; Li et al., 2021; Rodriguez et al., 2021). The fact that makes the immune system unique is the significant variety in the type of cells, with sometimes only few cells existing from one specialized lineage. This is exactly where a significant need for single-cell analysis is sensed, and single-cell sequencing both for DNA and RNA comes to the rescue. Accordingly, numerous groups have been focusing on single-cell analysis on pancreatic pathological conditions and discovering new immunological mechanisms. Kemp et al., for instance, fetched out a new way to molecularly detect pancreatic cancer, as they discovered special tumor-associated macrophages with the same traces present in the blood’s monocytes (Kemp et al., 2021). They analyze the relatively small number of monocyte cells present in the blood sample down to the transcriptome owing to single-cell RNA sequencing. As another example, the same group published a detailed descriptive paper on the molecular landscape of immune cells in the tumor and peripheral blood, again using single RNA sequencing (Steele et al., 2020). Notably, they attained a pool of genetic data out of which several biomarker candidates could be selected. As another creative and more combinatory study example, Sivakumar et al. (2021) incorporated mass cytometry, single-cell RNA sequencing, immunohistochemistry, and spatial analysis focusing on T cells to reconstruct the immune system microenvironment. Using this approach, they discovered that unlike CD4^+^ and CD8^+^ cells that were coherently spread in the tissue, regulatory T cells were meaningfully present only in the stromal cancer tissue with minimum presence in the epithelial compartments, at both cancer and normal sides. This was the piece of evidence for their hypothesis of activated Treg cells being in charge of PDAC progression. Also, they looked into the indifference of CD8^+^ T cells to the presence of a tumor and found senescence in charge, mostly analyzed by single-cell RNA sequencing.

## 5 Conclusion

Single-cell RNA sequencing is a powerful tool for studying pancreatic physiology and pathophysiology. However, there is a main difference in the way researchers utilized the technique. Some used complementary sorting methods, such as mass cytometry, to navigate the cellular population down to a more known subpopulation and then used sequencing. This methodology always provides easier analysis for the output sequencing data since we obtained good molecular clues for the sorted population. On the contrary, sequencing on unsorted tissue cells will produce sequenced data that are much harder to analyze but with much more novel information. In other words, completely new insights into biological mechanisms, such as new correlations, complex signaling networks between cells, or whole new lineages, must be extracted from a pool of raw sequenced data with all the subsets of the tissue present. Notably, pancreatic tissue is a relatively complex one, being involved in the diverse physiological processes of the complex endocrine, nervous, and digestive systems that ensures the complex unknown correlations between cells from different pancreas components, enlightening the prominence of the whole descriptive tissue single-cell study for the pancreatic, nervous, and endocrine biology. Also, a combination with other technologies such as microfluidics and nano-fabrication can always be beneficial as it has already provided us with significant inventory tools such as spatial transcriptomics. In other words, future single-cell studies on the pancreas have ways to improve, enabling us to perform single-cell sequencing for the whole tissue by scaling up the technology, also to be used in combination with other sorting methods for the whole tissue, not losing data of a single cell. The good news is that computers and the regrading algorithms have been developed in advance and are ready to satisfy the need for scaling up the transcriptomics. Finally, the prominent early result of these combinations seems to be an enhancement in spatial transcriptomics, improving its limitations to the accessible goal of single-cell spatial resolution and for the whole tissues. Overall, having explained the applications of single-cell sequencing and analysis in pancreatic studies, the journey of potential future applications seems to have just started, and a load of new insights will emerge with advances in novel instruments and methodologies.
